# Motion Estimation-Assisted Denoising for an Efficient Combination with an HEVC Encoder

**DOI:** 10.3390/s19040895

**Published:** 2019-02-21

**Authors:** Seung-Yong Lee, Chae Eun Rhee

**Affiliations:** Department of Information and Communication Engineering, Inha University, Incheon 402-751, Korea; ten1552@naver.com

**Keywords:** high-efficiency video coding (HEVC), video compression, denoising, block matching 3D collaborative filtering (BM3D)

## Abstract

Noise, which is commonly generated in low-light environments or by low-performance cameras, is a major cause of the degradation of compression efficiency. In previous studies that attempted to combine a denoise algorithm and a video encoder, denoising was used independently of the code for pre-processing or post-processing. However, this process must be tightly coupled with encoding because noise affects the compression efficiency greatly. In addition, this represents a major opportunity to reduce the computational complexity, because the encoding process and a denoise algorithm have many similarities. In this paper, a simple, add-on denoising scheme is proposed through a combination of high-efficiency video coding (HEVC) and block matching three-dimensional collaborative filtering (BM3D) algorithms. It is known that BM3D has excellent denoise performance but that it is limited in its use due to its high computational complexity. This paper employs motion estimation in HEVC to replace the block matching of BM3D so that most of the time-consuming functions are shared. To overcome the challenging algorithmic differences, the hierarchical structure in HEVC is uniquely utilized. As a result, the computational complexity is drastically reduced while the competitive performance capabilities in terms of coding efficiency and denoising quality are maintained.

## 1. Introduction

In various applications, the demand for high-resolution video content is steadily increasing. Ultra-high-definition (UHD) images have become more popular than high-definition (HD). In contrast, storage space and/or network bandwidth remain insufficient, and thus, video compression is essential. High-efficiency video coding (HEVC) was standardized in 2013 by the joint collaborative team on video coding (JCT-VC) and is now widely used for various applications. HEVC increases the computational complexity by about 1.5 times compared with the existing compression standard H.264, but the compression rate is nearly doubled [1,2,3,4]. This compression efficiency depends on how much the temporal and spatial correlation of the input video is utilized. Various coding tools have been improved or newly proposed to eliminate temporal and spatial redundancy in inter-prediction and intra-prediction. For example, merge mode has been proposed to save the bits used to code motion vector (MV) information. In merge mode, neighboring blocks having the same MV are merged to share the MV, thereby saving the bits used in MV coding. The fact that SKIP mode, which does not need to send encoding information when the quantized residual is zero, is applied to blocks of various sizes is different from the conventional H.264.

In certain devices, such as eye glasses, car black boxes, surveillance cameras, or badge cameras, the operation of a camera is necessary regardless of the time or place. Low-light environments or low-performance cameras often result in annoying noisy images [5,6,7]. This noise reduces the spatial and temporal redundancies of the video, thereby drastically reducing the compression efficiency. When applying denoising techniques to an encoder, both the image quality and the compression efficiency should be carefully considered. Table 1 shows the bitrates that increase when noise is added to the HEVC test sequences. The experimental environment is as follows. In the lowdelay_P_main configuration, 10 frames were encoded with quantization parameters (QP) 22, 27, 32, and 37 using HM-16.10 reference software [8]. Additive white Gaussian noise (AWGN) with sigma 20 was added to each input test sequence. The first and second columns of Table 1 indicate the size and name of each video. The third column shows the bitrate of clean video, whereas the fourth column shows the bitrate of noisy video. This is the average of the bitrates encoded with four QP values. When noise is added, each video shows an average bitrate increase of 876%. It can be seen that the temporal and spatial redundancy of the image is reduced due to the noise, and the compression efficiency is greatly reduced.

There have long been numerous studies of denoising methods. These can be divided largely into local and non-local approaches. Local schemes utilize neighboring pixels. Examples include the mean filter, Gaussian filter, and bilateral filter [9]. Meanwhile, non-local schemes use not only the surrounding data, but also the data of the entire image or neighboring frames. Thus, noise removal is more effective when using a non-local scheme than when using a local scheme. Non-local mean, block matching three-dimensional collaborative filtering (BM3D), and shape-adaptive discrete cosine transform (SA-DCT) are known to be the best denoising algorithms [10,11,12,13]. All these works are based on the observation that local image patches are often repetitive within an image. Similar patches are grouped and collaboratively filtered to remove noise. In video denoising [14,15,16], similar patches are searched and matched over time for noise removal to exploit redundant data further. Different from existing methods, the algorithm in [17] is derived with minimal assumptions on the statistical properties of image noise, where a denoising problem is converted from the stack of matched patches to a low rank matrix completion problem. Utilizing the characteristics of previous studies, recent works [18,19] have tried to apply different algorithms for dynamic foreground and static background. Deep learning approaches are also actively adopted to denoising algorithms [20]. In [21], an online block matching technique was proposed, and little memory use and efficient processing was achieved while keeping good denoising performance. However, the computational complexity associated with these methods is quite high due to the intensive search and block matching processes. Several studies have attempted to combine denoising and encoding. Techniques on the encoder side have focused on compression efficiency. A non-linear Crawford filter or a Gaussian filter can be used to denoise the input frame before encoding [22,23]. Here, the side information, which can be obtained from the encoding process, is not utilized. On the decoder side, for reconstructed videos, non-local similarity block prediction and temporal auto-regressive approaches can be used to improve image quality [24]. In other studies, the original image has been predicted using the inverse transform and predictions of the reconstructed video, thereby reducing the image quality degradation caused by lossy compression [25]. These decoder-side schemes mainly aim to improve image quality. However, the bitrate is greatly increased, because the noisy images should be compressed on the encoder side. In most previous approaches, video coding and denoising are performed independently, and the execution time is greatly increased as the computational complexity of the two processes is likewise increased.

This paper proposes a denoising scheme assisted by motion estimation (ME) combined with an HEVC video encoder. Denoise and encoding algorithms share the most time-consuming block matching process. More specifically, ME, repeatedly performed in a hierarchical structure, provides useful information for BM3D. The proposed combined scheme achieves denoising close to BM3D in the encoder while greatly reducing the computational complexity.

The rest of this paper is organized as follows. In Section 2, the combination of HEVC and BM3D is proposed. In Section 3, the performance is evaluated. Finally, Section 4 concludes the paper.

## 2. Proposed Video Denoising through the Combination with HEVC

### 2.1. Overview

In HEVC [26,27,28,29], a video frame is partitioned into multiple coding tree units (CTUs) as the basic processing unit. A CTU can be split, forming a quad-tree structure with a leaf on the tree, referred to as a coding unit (CU). For a CU of which the size is denoted as 2N × 2N, there are four types of prediction units (PUs): 2N × 2N, 2N × N, N × 2N, and N × N. Different predicted blocks are generated for different PUs, and then, either the inter- or intra-prediction mode is selected as the CU. The inter-prediction of the HEVC is an operation to find the block most similar to the current block through ME, and it is repeatedly performed at different CU depths. BM3D consists of two steps. In step 1, blocks similar to the current block are searched and grouped. Then, three-dimensional (3D) transform, collaborative filtering, and inverse transform are performed. The weight of each group is determined according to the filtering result. Denoised blocks are formed through the weighted summation of filtered blocks. In step 2, a similar process is performed again on the first denoised image to reduce the noise further.

This paper focuses on the fact that HEVC and BM3D have a block matching process in common. Block matching is one of the most time-consuming parts in both algorithms. Certain difficulties arise when replacing BM3D block matching directly with ME. First, the denoise process should take advantage of the ME results. However, for compression efficiency, denoised blocks should be used as the input for all the prediction processes. Second, in BM3D, a pixel-basis search algorithm is used in the current frame to obtain a rich overlapping block patch. However, the HEVC performs a non-overlapping search on a per-PU basis in the reference frame rather than in the current frame. Third, in BM3D, aggregation is applied to all the blocks that are searched. However, this does not comply with the HEVC standard, where the reference frames that are encoded and reconstructed cannot be modified. In this paper, these problems are overcome by making good use of the characteristics of the HEVC encoding process. First, the denoise function is inserted between integer ME (IME) and fractional ME (FME). IME replaces the block matching in BM3D. At the same time, the remaining encoding is performed with the denoised data. Second, the overlapping patch blocks are obtained in the depth hierarchy of the CTU. Utilizing this feature, only minor aggregation is performed. The aggregation result is not reflected in the reference frame.

**Figure 1:**
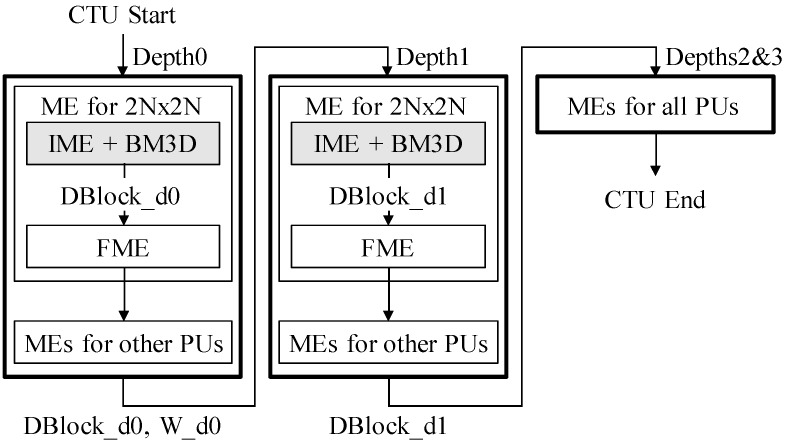
High-efficiency video coding (HEVC) encoding flow combined with block matching three-dimensional collaborative filtering (BM3D). CTU: coding tree units; ME: motion estimation; IME: integer ME; FME: fractional ME; and PUs: prediction units.

Figure 1 shows the overall flow of the proposed algorithm. In the gray shaded areas, the HEVC and simplified BM3D (sBM3D) algorithms are combined. sBM3D covers step 1 of BM3D. First, sBM3D is performed on the block patches obtained from the IME for the 2N × 2N PU of Depth0. Denoised *DBlock_d0* replaces the noisy input block, and FME of 2N × 2N PU is performed. Then, other PU types also use *DBlock_d0*. Intra-prediction is excluded from the figure for simplicity. When proceeding to Depth1, *DBlock_d0* and the corresponding weight *W_d0* are transmitted. The weight is determined by the number of coefficients that are the result of collaborative filtering. The larger the number of coefficients, the greater the weight, because there is a lot video information remaining. In Depth1, sBM3D is performed after the IME of 2N × 2N PU in a manner similar to that used with Depth0. At this time, *DBlock_d0* from Depth0 is added to the group. At this point, *DBlock_d1* is generated after aggregation using *W_d0* and *W_d1*. Depth2 and Depth3 perform all the predictions using recently denoised input blocks, *DBlock_d1*. There is no restriction on the block size. If the CU size is 8 × 8, the minimum unit of the inter block grouping can be performed. However, the advantage of depth hierarchy is not available. The proposed schemes are described in detail in the following sections.

### 2.2. Integer Motion Estimation-Based Grouping

**Figure 2:**
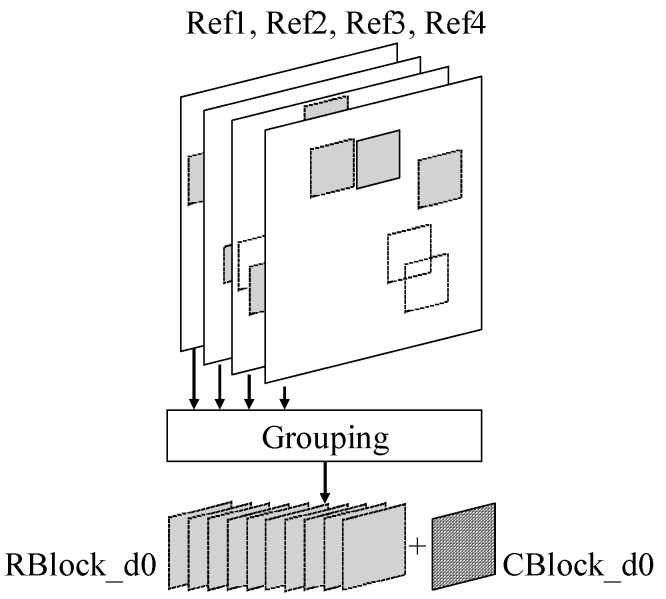
IME-based patch grouping.

Figure 2 shows the grouping process of sBM3D for a noisy 2N × 2N PU block in Depth0 using the IME results. In the ME of HEVC, block matching is performed only in the reference frame and not in the current frame. Nevertheless, it is possible to obtain an adequate number of similar block patches from the neighboring frames given that there is high temporal correlation between the frames. The noisy current block of Depth0 is denoted by *CBlock_d0*. IME is performed on the reference frames, Ref1, Ref2, Ref3, and Ref4, and then, grouping is performed with blocks having a sum of absolute difference (SAD) value smaller than *THgroup* among the searched blocks. This group is denoted by *RBlock_d0. CBlock_d0* is also added to the group *RBlock_d0*. *THgroup* is set to 1.2 times the value of *minSAD*, which is the smallest SAD value of the searched blocks. If only the blocks with *minSAD* are chosen, there will only be a small number of blocks to group. Blocks with a SAD value of 1.2 times the *minSAD* are determined to be sufficiently similar experimentally. Therefore, the selection of blocks corresponding to *minSAD* × 1.2 enables a sufficient group size. The CU size of HEVC varies from 64 × 64 to 8 × 8. If sBM3D is always performed with a size of 2N × 2N PU, it will be difficult to denoise precisely when the block size is large (e.g., 64 × 64). Therefore, *RBlock_d0* and *CBlock_d0* are divided into 8 × 8-sized units. Transforming and filtering (TF) processes are then conducted in the 8 × 8 units [30].

In the HM reference software, IME and FME are performed for each reference frame in turn. There are two problems when applying IME-based sBM3D directly to the existing encoding process. First, the number of patch blocks of the group is too small. Denoising should be performed using only the group obtained from the IME of Ref1 before FME of Ref1. Second, grouping, TF, and aggregation are repeated the same number of times as the number of reference frames, resulting in high computational complexity. Therefore, in this paper, each FME was performed after finishing the IME for all the reference frames. This modified sequence of procedures is commonly used to increase the parallelism of hardware-based ME [31]. At this stage, a sufficient number of blocks are obtained for grouping. The rest of the process of sBM3D, except for grouping, is performed only once regardless of the number of reference frames, thereby reducing the processing time.

### 2.3. Depth Hierarchy-Based Aggregation

**Figure 3:**
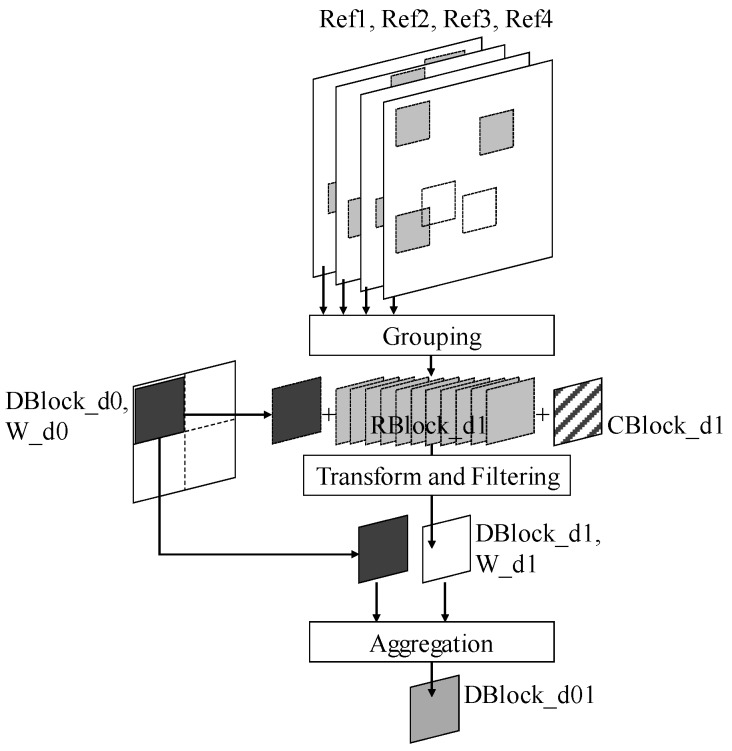
Overlapping patch acquisition and aggregation.

From Depth1, the hierarchical ME processing of HEVC is actively utilized. Figure 3 shows the sBM3D in a CU of Depth1. The noisy current 2N × 2N PU is denoted by *CBlock_d1*. *RBlock_d1* values for *CBlock_d1* are obtained from the reference frames using the scheme introduced in Section 2.2. *CBlock_d1* and *RBlock_d1* are grouped. The area corresponding to *CBlock_d1* is extracted from *DBlock_d0* and then added to the group. After performing TF, this group has a weight of *W_d1*. Next, aggregation is performed using *W_d0*, which is the weight from the upper depth. Thus, sBM3D at multiple depths can overcome some of the limitations of non-overlapping block matching. Several overlapping patch blocks naturally allow aggregation. In addition, a good block patch can be obtained by also including the denoised blocks of the upper depth in a group at a lower depth. Hence, effects similar to those in step 2 of BM3D can be expected. In this paper, sBM3D was performed only for Depth0 and Depth1. When sBM3D is performed for all depth CUs, the computational complexity increases sharply, whereas the additional denoising effect is insignificant.

### 2.4. Early Denoising Termination

If the characteristics of HEVC encoding are considered, the execution time of sBM3D can be reduced without significantly degrading the denoising performance. In HEVC encoding, after all of the depths are predicted, the best depth is selected to determine the partition of the CTU. In this case, if the upper depth is determined to be the best depth, the denoising process performed at the lower depth represents an inefficient use of time. The proposed early denoising termination (EDT) process is as follows. In Depth0, the prediction, as well as the proposed sBM3D, is performed. If the best prediction mode is determined to be the SKIP or merge mode of 2N × 2N, meaning that a prediction is achieved feasibly considering the rate distortion (RD) when using a properly denoised block, Depth1 does not perform an additional denoising process but uses the denoised block from Depth0 as is. If the best prediction mode of Depth0 is not SKIP or 2N × 2N merge, sBM3D is performed in Depth1, as explained in Figure 3.

## 3. Performance Evaluation

To evaluate the performance of the proposed scheme, eight sequences of various sizes were used. All the experiments were conducted on an Intel i7-8700K running 3.70 GHz and 64 GB memory. Noise was added using the AWGN of sigma values of 15 and 25. Each sequence consisted of 50 frames. The low-delay-P-Main (LD) configuration provided in HM-16.10 was used unchanged with four depths, where 64 × 64 and 32 × 32 blocks are used as coding units for depths 0 and 1, respectively. The proposed scheme can be used along with all the encoding modes except all-intra, which does not use motion estimation. A basic test zone (TZ) search algorithm was used from the HM-16.10 reference software. Fast and smart search algorithms can quickly get good candidates for denoising [32,33,34,35,36,37]. Moreover, the quality of patches is generally more important than the number of patches. In the BM3D configuration, the maximum number of patches is usually set to 16. Therefore, the proposed scheme was not significantly affected by the search algorithm. QPs were set to 22, 27, 32 and 37. Four conventional denoising algorithms were used for a performance comparison. For BM3D, Marc Lebrun’s open-source code was used [16]. In step 1 of BM3D, the block size was 8 × 8 pixels, and the target number of block patches was 16. The search area was 32 pixels horizontally and vertically with a spacing distance of three pixels. In step 2 of BM3D, the target number of block patches was set to 32. For a simplified but rapid BM3D version, only step 1 was performed where the block size was 16 × 16 pixels and the target number of block patches was 16. The search area was 16 pixels horizontally and vertically with a 16-pixel spacing distance for rapid processing. The Gaussian filter used sigma 1, whereas the bilateral filter adopted a 5 × 5 filter size with sigma 1. Hereafter, the encodings which use BM3D, simplified BM3D, a Gaussian filter, and a bilateral filter as pre-processing are referred to as *preBM*, *preBMF*, *preG*, and *preBi*, respectively. *IBM* refers to a scheme in which sBM3D is independently applied to Depth0 and Depth1 through the proposed IME-based grouping, whereas *IHBM* means that sBM3D utilizes hierarchical information. The denoised blocks at Depth0 were divided into four parts and transmitted to Depth1, and filtering was performed once again. When the EDT scheme was additionally applied in Depth0, it was denoted by *IEHBM*.

Table 2 shows the compression efficiency and denoise performance when using the conventional denoising algorithms and the proposed methods. First, the compression efficiency was measured with the Bjøntegaard delta bit rate (BDBR) [38], which was calculated using the bitrate used during the transmission and the peak-signal-to-noise ratio (PSNR) between the encoder input and the reconstructed images. The BDBR numbers above the dotted lines in Table 2 show the improved compression efficiency when denoising was applied compared with encoding the noisy frames. *preBM* showed the best compression efficiency, whereas *preBi* showed the lowest efficiency. Regardless of the quality of the denoised image, *preG* exhibited denoising results closest to *preBM* in terms of the compression efficiency. The proposed schemes, *IBM*, *IHBM*, and *IEHBM*, showed the next best denoising performance. Note that if noise reduction is over-applied and the image is blurred, the compression efficiency would be very good. If a blurred image is encoded, a much improved BDBR value can be obtained. Therefore, in order to evaluate both compression efficiency and denoising performance, the quality of the reconstructed denoised frame should be measured along with the encoding efficiency. For this, the denoise performance of each algorithm is represented by a PSNR, which are presented below the dotted lines in Table 2. The difference between two reconstructed images was calculated for a case in which a clean original image without noise was encoded and a case in which a noisy image was denoised and then encoded. The PSNR was the average value when four QPs used in the BDBR measurement were applied. When the noisy video with a sigma = 15 were encoded using a LD and random-access-main (RA) mode, *preBM* had the highest PSNR and *preG* has the lowest value. The Gaussian filter of *preG* blurred the image excessively. Therefore, the compression efficiency was high, but the denoising image quality became low. The proposed *IBM*, *IHBM*, and *IEHBM* methods offered denoise performances similar to that of *preBMF*. *IHBM* was superior to *IBM* in terms of both the compression efficiency and denoising performance when using the depth hierarchy. *IEHBM*, created by modifying *IHBM* to use only the limited depth hierarchy, showed a slight reduction in the compression efficiency. When pre-denoised images were encoded with a RA mode, the BDBR was enhanced a little bit compared with a LD mode. However, the proposed schemes showed a similar level of BDBR values because denoising was performed independently for different prediction directions in the current implementation. If patches from different directions are grouped together, better compression efficiency is expected. When sigma = 25 in LD mode, the compression efficiency was enhanced compared with sigma = 15 in LD mode for the proposed schemes. The performance of *preG* and *preBi* was significantly reduced in terms of both compression efficiency and the quality of reconstructed frame.

Table 3 shows the processing time required for denoising and encoding when sigma = 15 in LD mode. The numbers above the dotted lines represent the total processing time, whereas the numbers below the dotted lines represent the denoising time. Subtracting the denoising time from the total processing time results in the encoding time. *preBM* has the longest time due to its high computational complexity, whereas the execution time of *preG* is the shortest. Compared with *preBM*, the proposed *IBM*, *IHBM*, and *IEHBM* methods showed a reduction in the execution time of around 40%. *IBM*, which does not use depth hierarchy information, showed the shortest processing time among the proposed schemes. *IHBM* has the longest processing time, because it performs denoising and encoding using the depth hierarchy for both Depth0 and Depth1. Because IEHBM selectively uses the depth hierarchy, it showed a reduction in the processing time of 11.7% per frame as compared to that by *IHBM*.

**Figure 4:**
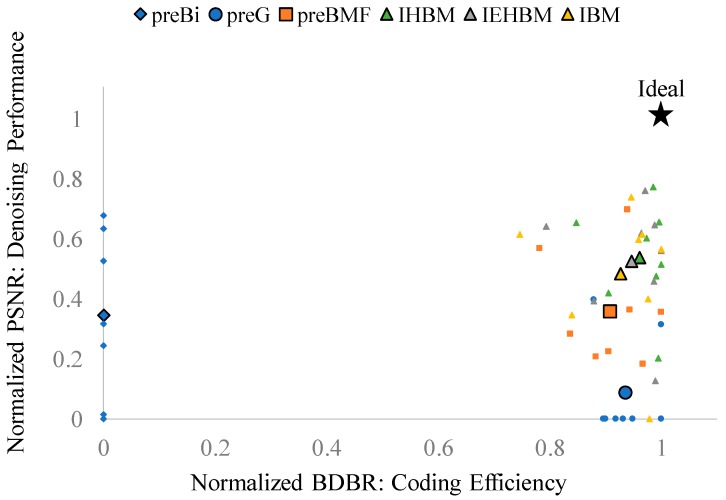
Compression–Denoising Performance.

Figure 4 shows the compression–denoising (C-D) performance outcomes for each algorithm. On the horizontal and vertical axes, BDBR and PSNR for sigma = 15 in the LD condition of Table 2 were normalized, respectively. The ideal point is indicated using the star symbol. *preBM* was assumed to be ideal and had a coordinate value of (1, 1). The coding efficiency of the denoised frame with *preBM* was best. The PSNR of the reconstructed frame was also the highest when compared with the original frame without noise. If the denoising schemes are located close to the upper-right corner, it can be said that they are competitive compared with *preBM* considering both coding efficiency and denoising performance. The eight small markers per algorithm were the result of eight test sequences. Because of the different characteristics of each video, the points were scattered but the overall trend could be grasped. For ease of comprehension, the average values of the eight test sequences conducted for each algorithm are denoted by large black bordered markers. The algorithm located closest to the upper-right corner of the coordinate space has the best C-D performance. *preBi* is at the leftmost position due to its low coding efficiency, whereas *preG* is located at the bottom, because its denoising performance is low. In most of the test sequences, the proposed schemes, denoted by triangles, indicate high C-D performance. Among them, *IHBM*, denoted by the green triangle, is located at the upper-rightmost position and shows the C-D performance closest to that of *preBM*.

**Figure 5:**
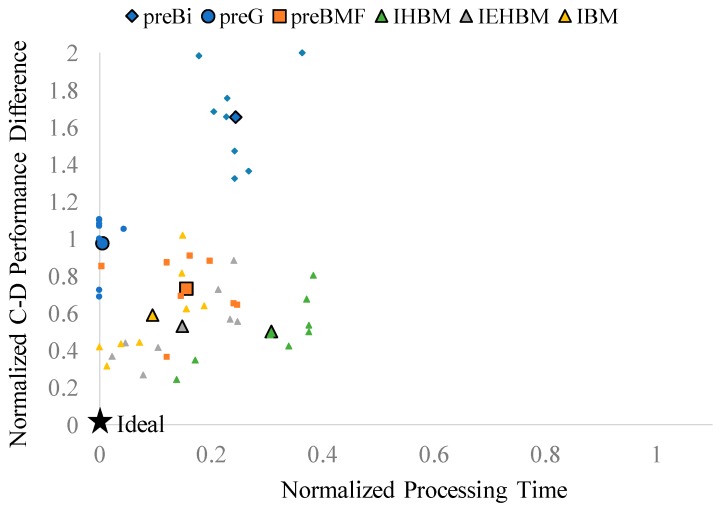
Compression–Denoising–Time Performance.

Figure 5 shows the compression–denoising–time (C-D-T) performance outcomes for each algorithm for sigma = 15 in the LD condition. The horizontal axis represents the processing time, which was normalized from Table 3. The vertical axis represents the difference in the C-D performance compared with *preBM*. The coordinates of the ideal point are (0, 0). When the horizontal axis is 0, the processing time becomes smaller than the *preBM* located at the horizontal axis = 1, and thereby, the computational complexity is reduced. On the vertical axis, which indicates the difference in performance from *preBM*, the C-D performance is excellent when the coordinate value is close to zero. How close the C-D performance of the algorithms is to that of *preBM* is measured by calculating the Manhattan distance between the coordinates of each algorithm and the (1, 1) position of *preBM* in Figure 4. The algorithm located at the bottom left of the coordinate space has the best C-D-T performance. *preBi* and *preG* are located at the top of the coordinate space due to their low C-D performances. *IHBM*, denoted with the green triangle, shows a somewhat longer processing time. *IEHBM*, denoted by the gray triangle, shows the best results on average when considering both the C-D performance and the processing time.

## 4. Conclusions

Although denoising algorithms and video encoding are typically used in conjunction with each other, a combination of the two algorithms has not been studied intensively. This paper analyzed the algorithms of HEVC and BM3D and proposed a competitive combination solution in terms of the coding efficiency, processing time, and denoising quality. In particular, the most time-consuming part, block matching, was shared so that both algorithms could be smoothly fused into one refined design. Moreover, to overcome the challenging algorithmic differences between BM3D and HEVC, a depth hierarchy was utilized to acquire more patches and to enable aggregation. Further research is needed to apply the proposed schemes to hardware-based encoders for more practical use.

## Figures and Tables

**Table 1:** Comparison of processing time for noisy video sequences.

Video Sequence		Bitrate (Mbps)	
		Clean	Noisy
		video	video
ClassB	Kimono	3.89	78.28
	BasketballDrive	7.34	163.54
ClassC	BasketballDrill	2.17	33.07
	BQMall	3.05	40.64
ClassD	BasketballPass	0.61	8.37
	BQSquare	1.63	10.48
ClassE	FourPeople	2.39	87.72
	Johnny	1.76	85.98
Average		3.18	30.98

**Table 2:** Comparison of coding efficiency and denoising performance for noisy video sequences. BDBR: Bjøntegaard delta bit rate; PSNR: peak-signal-to-noise ratio.

VideoSequence		BDBR (%)																				
		PSNR (dB)																				
		σ = 15 LD							σ = 15 RA							σ = 25 LD						
		preBi	preG	preBM	preBMF	IBM	IHBM	IEHBM	preBi	preG	preBM	preBMF	IBM	IHBM	IEHBM	preBi	preG	preBM	preBMF	IBM	IHBM	IEHBM
ClassB	Kimono	−80.9	−96.8	−99.0	−97.9	−98.6	−98.8	−98.9	−83.6	−97.5	−99.3	−98.5	−98.8	−99.1	−99.0	−76.4	−94.0	−98.9	−98.9	−99.0	−99.1	−99.0
		34.4	35.4	37.0	36.2	34.4	34.9	34.7	35.4	36.2	37.7	37.0	35.7	36.1	36.0	29.9	32.3	34.9	35.0	31.9	32.5	32.3
	BasketballDrive	−78.0	−97.0	−99.2	−98.0	−98.7	−98.9	−99.0	−81.2	−97.9	−99.5	−98.9	−99.2	−99.3	−99.3	−54.1	−93.9	−99.0	−99.0	−98.8	−99.0	−99.0
		34.2	32.7	37.3	34.4	34.6	34.9	34.8	35.4	33.3	38.3	34.8	35.0	35.5	35.4	29.4	30.8	35.3	35.3	31.6	32.5	32.0
ClassC	BasketballDrill	−74.7	−96.2	−97.8	−95.1	−94.1	−95.0	−95.6	−78.0	−97.1	−98.4	−96.3	−94.6	−95.8	−95.5	−50.7	−93.9	−97.4	−96.6	−96.5	−97.4	−97.0
		32.3	30.2	34.2	31.1	31.6	31.9	31.8	33.1	30.4	34.5	31.2	32.4	32.6	32.5	27.8	28.9	32.2	32.1	29.7	29.7	29.7
	BQMall	−70.4	−96.0	−96.0	−90.4	−89.5	−90.7	−92.1	−74.2	−97.1	−97.2	−92.1	−89.2	−91.0	−90.3	−47.3	−92.7	−95.8	−95.6	−94.5	−96.1	−95.3
		30.7	27.3	32.3	30.1	30.4	30.6	30.5	31.5	27.5	32.7	30.5	31.1	31.3	31.3	26.5	26.6	30.1	29.9	28.1	28.1	28.1
ClassD	BasketballPass	−74.3	−96.4	−97.6	−93.8	−96.8	−97.3	−97.5	−77.1	−97.1	−98.1	−94.8	−96.7	−97.3	−97.2	−50.5	−93.1	−97.3	−97.1	−98.0	−98.5	−98.3
		31.7	28.9	33.4	30.1	31.6	31.8	31.8	32.4	29.2	33.6	30.5	32.4	32.5	32.5	26.4	27.9	31.2	31.0	29.5	29.5	29.5
	BlowingBubbles	−40.6	−94.6	−93.6	−94.6	−91.7	−93.8	−93.0	−45.9	−96.2	−95.2	−89.2	−91.6	−93.5	−92.9	−46.2	−91.8	−94.5	−94.3	−96.3	−97.2	−96.7
		26.5	27.9	31.0	28.1	29.8	30.0	29.9	27.0	27.9	31.3	28.5	30.6	30.8	30.8	26.3	27.1	29.0	29.0	27.6	27.6	27.6
ClassE	FourPeople	−77.2	−97.5	−99.3	−97.2	−98.4	−98.5	−98.7	−79.8	−98.2	−99.5	−98.0	−97.7	−98.1	−97.9	−52.4	−94.0	−98.6	−98.6	−99.1	−99.2	−99.1
		33.3	31.9	36.1	32.8	34.4	34.4	34.5	34.2	32.1	36.6	33.1	35.0	35.1	35.1	28.3	30.2	33.7	33.7	32.0	32.0	32.0
	Johnny	78.9	97.7	99.8	99.1	99.8	99.8	99.8	−81.7	−98.4	−99.9	−99.5	−99.9	−99.9	−99.9	−54.9	−94.4	−99.5	−99.5	−99.8	−99.8	−99.8
		34.8	33.6	38.4	34.5	36.3	36.1	36.3	35.9	34.0	39.0	34.8	37.4	37.3	37.4	29.7	31.4	36.3	36.2	34.1	34.1	34.1
Average		−71.9	−96.5	−97.8	−95.8	−96.0	−96.6	−96.8	−75.2	−97.4	−98.4	−95.9	−96.0	−96.8	−96.5	−54.1	−93.5	−97.6	−97.5	−97.8	−98.3	−98.0
		32.2	31.0	35.0	32.2	32.9	33.1	33.0	33.1	31.3	35.5	32.5	33.7	33.9	33.9	28.0	29.4	32.8	32.8	30.5	30.7	30.7

**Table 3:** Comparison of processing time for noisy video sequences.

Video Sequence				Total Time/Frame (sec)				
				Denoising Time/Frame (sec)				
		preBi	preG	preBM	preBMF	IBM	IHBM	IEHBM
ClassB	Kimono	45.63	39.08	75.79	43.53	44.59	53.16	47.93
		0.20	0.05	43.50	7.30	6.70	14.50	9.80
	BasketballDrive	45.67	38.23	74.54	43.58	43.91	51.89	47.25
		0.20	0.05	42.40	7.30	8.10	14.10	9.80
ClassC	BasketballDrill	8.50	6.80	13.81	7.94	7.83	9.41	8.30
		0.04	0.01	7.70	1.20	1.70	2.50	2.00
	BQMall	8.38	6.61	13.88	8.37	7.97	9.35	8.32
		0.04	0.01	7.90	1.20	1.50	2.40	1.50
ClassD	BasketballPass	1.88	1.47	3.24	1.75	1.38	1.70	1.43
		0.01	0.00	2.00	0.30	0.40	0.60	0.50
	BlowingBubbles	2.39	1.70	3.59	2.17	1.73	1.96	1.85
		0.01	0.00	1.80	0.30	0.30	0.40	0.30
ClassE	FourPeople	16.86	13.31	28.89	15.21	14.44	18.62	14.95
		0.10	0.02	18.30	2.70	2.90	5.70	3.50
	Johnny	17.05	13.73	28.19	13.78	14.28	18.16	14.41
		0.10	0.02	18.60	2.70	2.90	6.10	3.10
Average		18.30	15.11	30.24	17.04	17.02	20.53	18.06
		0.09	0.02	17.78	2.88	3.06	5.79	3.81
